# Machine learning–based prediction of IVF/ICSI outcomes in male factor infertility highlighting couple-level BMI

**DOI:** 10.3389/fendo.2026.1772106

**Published:** 2026-02-10

**Authors:** Hu Li, Jie Gao, Yiran Li

**Affiliations:** 1 Shanghai Key Laboratory of Maternal Fetal Medicine, Shanghai Institute of Maternal-Fetal Medicine and Gynecologic Oncology, Shanghai First Maternity and Infant Hospital, School of Medicine, Tongji University, Shanghai, China; 2 Centre for Assisted Reproduction, Shanghai Key Laboratory of Maternal Fetal Medicine, Shanghai Institute of Maternal-Fetal Medicine and Gynecologic Oncology, Shanghai First Maternity and Infant Hospital, School of Medicine, Tongji University, Shanghai, China

**Keywords:** body mass index, clinical pregnancy, lightGBM, machine learning, male infertility

## Abstract

**Background:**

Most clinical prediction models for assisted reproductive technology focus primarily on female ovarian reserve markers and often under-represent male factors and the metabolic status of both partners. Additionally, traditional parametric models may have limited ability to capture nonlinear patterns within reproductive data. This study aimed to develop and validate a machine learning (ML)–based model to predict clinical pregnancy outcomes in couples with male factor infertility undergoing IVF/ICSI, and to explore model interpretability using Shapley Additive exPlanations (SHAP).

**Methods:**

This retrospective study analyzed 2,565 couples undergoing their first IVF/ICSI cycle for male factor infertility at Shanghai First Maternity and Infant Hospital between 2019 and 2025. The cohort was partitioned according to embryo transfer date, with the first 70% of cases assigned to the training set and the remaining 30% reserved as an temporal internal validation set. Feature selection was conducted using LASSO regression within the training set. Seven ML models, including LightGBM and Logistic Regression, were developed and optimized through 5-fold cross-validation. Model performance was evaluated using the area under the curve (AUC), accuracy, Brier score, and decision curve analysis. SHAP was employed to provide a visual interpretation of the optimal model.

**Results:**

Five predictors were selected in the training set: female BMI, male BMI, basal FSH, AMH, and female age. In the temporal validation set, all models demonstrated comparable discriminative performance (AUC range: 0.840–0.857). LightGBM achieved an AUC of 0.857 (95% CI: 0.830–0.882), with an accuracy of 0.775 and specificity of 0.909. DeLong tests indicated no statistically significant differences in AUC between LightGBM and Random Forest (P = 0.918), XGBoost (P = 0.985), or logistic regression (P = 0.067). Based on its overall stability across discrimination, calibration (Brier score = 0.145), and clinical utility, LightGBM was selected for interpretability analysis.

**Conclusions:**

A LightGBM-based prediction model demonstrated reasonable performance for predicting IVF/ICSI outcomes in couples with male factor infertility. Within this dataset, couple-level metabolic features were strongly associated with model predictions alongside traditional ovarian reserve markers. These findings reflect predictive associations rather than causal effects and suggest that metabolic characteristics may warrant consideration in risk stratification and counseling. Prospective studies are needed to determine whether targeted interventions can improve clinical outcomes.

## 1 Introduction

Infertility has become a global public health concern and affects approximately 15% of couples of reproductive age (1, 2). Male factors account for 40%–50% of these cases (3). For patients with severe oligozoospermia, asthenozoospermia, or teratozoospermia, *in vitro* fertilization (IVF) and intracytoplasmic sperm injection (ICSI) remain the most effective treatment options (4, 5). Nevertheless, despite advances in assisted reproductive technology, the clinical pregnancy rate per IVF/ICSI cycle is still only 40%–60% (6–8). Failed cycles place a substantial financial burden on patients and are frequently associated with considerable psychological distress (9, 10). Accordingly, accurate pre-treatment prediction of pregnancy success is important for individualized treatment planning and for setting realistic expectations.

Traditional prediction models, including the Templeton model and the Nelson model, are largely based on logistic regression (11–13). Although these models have broad applicability in general populations, they have several limitations. First, these models largely center on female age and ovarian reserve markers such as AMH and FSH, while giving limited consideration to partner-related characteristics and their potential interactions, including male BMI and age (14). Second, although logistic regression is a type of generalized linear model, prespecified regression models may still be limited in their ability to flexibly capture complex nonlinear relationships and high-dimensional structures that are commonly observed in reproductive datasets, unless nonlinear terms or interactions are explicitly modeled (15). Third, generic models are often not tailored to the male factor infertility subgroup (16), which can compromise predictive accuracy in this population.

In recent years, rapid progress in artificial intelligence and machine learning (ML) has introduced new approaches to clinical prediction. Compared with traditional statistical methods, ML algorithms—including random forest and gradient boosting trees—offer advantages in modeling complex, nonlinear, and high-dimensional data (17, 18). Prior studies have reported promising performance of ML approaches in polycystic ovary syndrome (PCOS) (19). However, high-precision ML models based on large samples and incorporating couple-level characteristics remain limited in the male factor infertility population. In addition, many ML models are considered “black boxes” with limited clinical interpretability, which hinders their broader implementation in practice (20, 21).

This study aims to develop an IVF/ICSI pregnancy outcome prediction model for couples with male factor infertility using a single-center, large-sample retrospective dataset and multiple ML algorithms. Particular attention is given to quantifying the contribution of spousal BMI within the prediction framework using SHAP analysis. The findings may offer additional insight to support clinical decision-making in this setting.

## 2 Materials and methods

### 2.1 Subjects and design

This retrospective cohort study used data from couples who underwent IVF or ICSI at the Reproductive Medical Center of Shanghai First Maternity and Infant Hospital between January 2019 and January 2025. This study was approved by the Research Ethics Committee of Shanghai First Maternity and Infant Hospital (KS25468). The inclusion criteria were: (1) a primary diagnosis of male factor infertility, including oligozoospermia, asthenozoospermia, or teratozoospermia, defined according to the WHO 5th edition criteria (22); (2) treatment with conventional IVF or ICSI; (3) complete follow-up records for pregnancy outcomes; (4) only the first IVF/ICSI treatment cycle was included for each couple. The exclusion criteria were: (1) severe uterine malformations or intrauterine adhesions in the female partner; (2) chromosomal karyotype abnormalities in either partner; (3) cycles involving donor sperm or oocytes; and (4) missing values in non-imputable administrative or eligibility variables (none in the final analytic cohort). Ultimately, 2,565 couples were included. To enhance the methodological rigor and better reflect real-world clinical application, the cohort was partitioned strictly according to the date of embryo transfer (23). The earliest 70% of cases (n = 1,797) were assigned to the training set, and the most recent 30% (n = 768) were reserved as an internal validation set. The training set was used exclusively for feature selection, model development, and hyperparameter tuning, whereas the validation set was used only for final model evaluation.

### 2.2 Data collection

Variables were extracted from the electronic medical record (EMR) system. Demographic characteristics included female age, male age, female body mass index (BMI), male BMI, infertility duration, infertility type, female education, and male education. Clinical characteristics included menstrual regularity; basal follicle-stimulating hormone (FSH), luteinizing hormone (LH), estradiol (E2), progesterone (P), testosterone (T), prolactin (PRL), and anti-Müllerian hormone (AMH). The outcome was clinical pregnancy, defined as the presence of a gestational sac with fetal cardiac activity in the uterine cavity on transvaginal ultrasound 28–35 days after embryo transfer (24). Absence of a gestational sac or biochemical pregnancy was classified as non-clinical pregnancy.

### 2.3 Data preprocessing and feature selection

For missing data, multiple imputation was performed using the mice package in R (25). In this dataset, all candidate predictors exhibited low levels of missingness (<5% in both the training and validation sets; **Supplementary Table S1** and **Supplementary Figure S1**) (25). No participants were excluded due to missing non-imputable administrative or eligibility-defining variables (e.g., outcome follow-up), and therefore the final analytic cohort contained complete information on all eligibility-defining variables. Given the low proportion of missingness across predictors, multiple imputation was applied to all predictors to avoid unnecessary case deletion while minimizing potential instability associated with highly incomplete variables. Five imputed datasets were generated (m = 5, seed = 123). To avoid information leakage, imputation was conducted after the temporal split and performed separately within the training set and the temporal validation set. In the training set, the imputation model included all candidate predictors and the outcome variable in order to preserve predictor–outcome associations for model development. In the validation set, the imputation model included only predictors and explicitly excluded the outcome variable, thereby preventing outcome-informed imputation during model evaluation. Predictive mean matching was used for continuous variables and logistic regression for binary variables, with 20 iterations per imputation. The predictor matrix followed the default mice setting in which predictors were allowed to inform each other, except where structurally inappropriate; specifically, the outcome variable was excluded from all validation-set imputation models, and administrative or eligibility-defining variables were not imputed. The full imputation methods and predictor matrix for the training set are reported in **Supplementary Table S2** and **Supplementary Figure S2**. Feature selection was performed using the least absolute shrinkage and selection operator (LASSO) within the training set only (26). LASSO was implemented in R using the glmnet package with internal standardization (standardize = TRUE). LASSO was fitted separately within each of the five imputed training datasets, and predictors were retained in the final feature set if they showed stable selection at λ_1_SE across imputations, operationalized as non-zero coefficients in at least four of the five imputed datasets (27). The penalty parameter λ was selected using 10-fold cross-validation, and the minimum deviance occurred at λ_min = 0.00406, while the 1-standard-error criterion selected λ_1SE = 0.01801. For downstream machine learning analyses, continuous predictors were standardized to Z-scores using the StandardScaler function in the Python scikit-learn library (28), with scaling parameters learned from the training set and then applied to the validation set. Model development proceeded separately within each imputed training dataset. Each fitted model was then applied to each imputed validation dataset to generate predicted probabilities; for each individual, predicted probabilities were averaged across imputations to obtain pooled predictions. All performance metrics were calculated using these pooled predicted probabilities.

### 2.4 Model construction and hyperparameter tuning

Seven commonly used machine learning algorithms were developed to predict pregnancy outcomes: logistic regression (LR), decision tree (DT), random forest (RF), support vector machine (SVM), artificial neural network (ANN), XGBoost, and LightGBM. Hyperparameters for each model were optimized using 5-fold cross-validation with grid search within the training set. To ensure comparability and avoid optimistic bias under multiple imputation, hyperparameter tuning was performed only once using the first imputed training dataset, with a fixed random seed (seed = 123) (29). The resulting optimal hyperparameters were then held constant and applied to all five imputed training datasets for model fitting. This strategy ensured that model complexity and tuning degrees of freedom were consistent across imputations while allowing uncertainty due to imputation to be reflected in model estimation.

### 2.5 Model evaluation and interpretation

Model performance was evaluated in the validation set using the area under the receiver operating characteristic curve (AUC), accuracy, sensitivity, specificity, positive predictive value (PPV), negative predictive value (NPV) and F1 score. For all classification metrics, predicted probabilities were dichotomized using a fixed operating threshold of 0.5, which was applied consistently across all models and datasets to ensure comparability.

Calibration was evaluated using the Brier score (30) and calibration curves constructed by grouping predicted probabilities into deciles and plotting observed versus predicted outcome probabilities. Clinical utility was examined using decision curve analysis (DCA) over a clinically plausible threshold probability range of 20–80%, reflecting the range in which clinicians may reasonably consider counseling or intervention in the context of IVF/ICSI outcome prediction.

Models were fitted separately within each of the five imputed datasets. For each individual and each model, predicted probabilities were averaged across imputations to obtain a single pooled predicted probability. All discrimination metrics (including AUC), classification metrics, calibration analyses, and decision curve analyses were computed based on these pooled predicted probabilities.

The 95% confidence intervals for AUC were estimated using nonparametric bootstrap resampling (1,000 replications) applied to the pooled predicted probabilities within each dataset. Pairwise comparisons of AUCs between models were conducted using DeLong’s test for correlated receiver operating characteristic curves based on ROC curves constructed from the pooled predicted probabilities. Specifically, the AUCs of each model were compared against those of LightGBM and logistic regression, respectively, and the corresponding P values were reported.

The representative model was then interpreted using SHAP to quantify the marginal contribution of each feature (31). SHAP summary plots, beeswarm plots, and dependence plots were generated to illustrate the model’s decision patterns. SHAP values were computed using the standard interventional SHAP implementation provided by the SHAP Python package. We acknowledge that when predictors are correlated, feature attributions may be influenced by feature dependence, and therefore SHAP results should be interpreted as model-based associations rather than causal effects.

### 2.6 Statistical analysis

All analyses were performed using R (version 4.2.0) and Python (version 3.9.0). In R, data preprocessing, multiple imputation, descriptive analyses, dataset partitioning, and LASSO feature selection were conducted using standard statistical packages, including mice, caret, tableone, and glmnet. All machine learning model development, hyperparameter tuning, and performance evaluation were conducted in Python. Graphical analyses, including ROC curves, calibration curves, and decision curve analysis, were generated using matplotlib and ggplot2.

## 3 Results

### 3.1 Baseline characteristics

Baseline characteristics of the total cohort, training set, and validation set are presented in **Table 1**. The distributions of outcomes, demographic variables, and clinical characteristics were highly comparable between the training and validation sets. All standardized mean differences (SMDs) were below 0.25, with most variables showing SMDs < 0.10, indicating good balance between the two datasets. Slight imbalances were observed for female age (SMD = 0.218) and AMH (SMD = 0.113), but the overall clinical characteristics remained broadly similar across the two subsets, supporting the appropriateness of the temporal split for model development and validation.

**Table 1:** Baseline characteristics of the overall cohort and comparison between the training and validation sets.

Characteristics	Total (n=2565)	Training set (n=1797)	Validation set (n=768)	*SMD^1^*
Outcomes (%)				0.06
No Clinical pregnancy	1413 (55.09)	974 (54.20)	439 (57.16)	
Clinical pregnancy	1152 (44.91)	823 (45.80)	329 (42.84)	
female_education (%)				0.078
lower education level	624 (24.33)	455 (25.32)	169 (22.01)	
higher education level	1941 (75.67)	1342 (74.68)	599 (77.99)	
male_education (%)				0.066
lower education level	618 (24.09)	448 (24.93)	170 (22.14)	
higher education level	1947 (75.91)	1349 (75.07)	598 (77.86)	
infertility_type (%)				0.057
primary infertility	1695 (66.08)	1173 (65.28)	522 (67.97)	
secondary infertility	870 (33.92)	624 (34.72)	246 (32.03)	
menstrual_pattern (%)				0.002
regular menstrual cycle	1869 (72.87)	1309 (72.84)	560 (72.92)	
irregular menstrual cycle	696 (27.13)	488 (27.16)	208 (27.08)	
female_age	32.00 (30.00, 35.00)	32.00 [29.00, 34.00]	33.00 [30.00, 35.00]	0.218
male_age	33.00 (31.00, 36.00)	33.00 [31.00, 36.00]	34.00 [31.00, 37.00]	0.159
female_bmi	21.70 (19.50, 24.10)	21.60 [19.50, 24.10]	21.75 [19.58, 24.20]	0.076
male_bmi	25.10 (21.40, 28.30)	25.10 [21.40, 28.10]	25.20 [21.37, 28.63]	0.048
infertility_duration	2.10 (1.10, 4.00)	2.00 [1.00, 4.00]	2.20 [1.20, 4.00]	0.038
AMH	3.71 (2.13, 6.01)	3.74 [2.16, 6.21]	3.64 [1.93, 5.80]	0.113
FSH	6.53 (5.27, 7.84)	6.53 [5.28, 7.76]	6.56 [5.27, 8.18]	0.029
LH	4.19 (2.94, 5.82)	4.20 [2.91, 5.94]	4.19 [3.02, 5.80]	0.051
PRL	12.00 (8.85, 16.74)	12.35 [9.05, 17.60]	11.91 [8.73, 16.39]	0.078
E2	43.00 (32.00, 62.95)	43.33 [32.63, 63.73]	42.00 [31.20, 62.74]	0.002
T	0.26 (0.20, 0.37)	0.27 [0.20, 0.37]	0.25 [0.19, 0.36]	0.053
P	0.58 (0.42, 0.79)	0.58 [0.42, 0.79]	0.57 [0.41, 0.80]	0.077
AFC_total	17.00 (12.00, 21.00)	17.00 [12.00, 21.00]	17.00 [11.75, 21.00]	0.03

^1^Standardized mean difference.

Data are shown as median with interquartile range (IQR) for continuous variables and number with percentage for categorical variables.

AMH, anti-Müllerian hormone; FSH, follicle-stimulating hormone; LH, luteinizing hormone; PRL, prolactin; E2, estradiol; T, testosterone; P, progesterone; AFC_total, total antral follicle count.

### 3.2 LASSO regression feature selection

**Figure 1:**
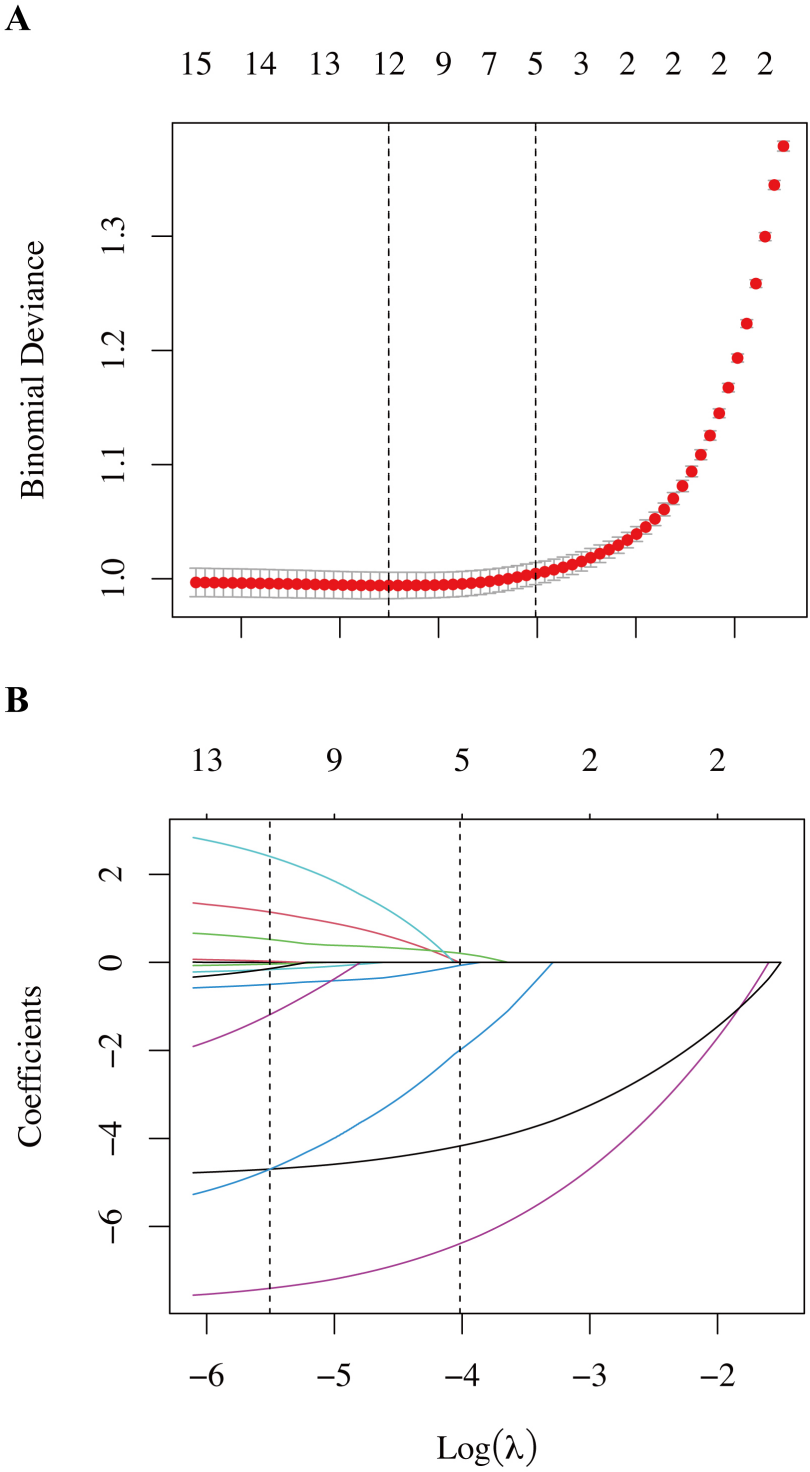
Feature selection using the LASSO regression model. **(A)** LASSO Regression Model Factor Selection: Left dashed line represents the optimal lambda value (lambda_min), while the right dashed line marks the lambda value within one standard error of the optimal (lambda.1se). **(B)** LASSO regression model screening variable trajectories.

LASSO regression was applied to reduce multicollinearity and identify key predictors. As shown in **Figures 1A, B**, the penalty parameter was selected using 10-fold cross-validation. The minimum cross-validated deviance occurred at λ_min = 0.00406, while the 1-standard-error criterion selected λ_1SE = 0.01801, yielding a parsimonious set of five predictors with non-zero coefficients. Across the five imputed training datasets, five predictors were consistently selected by LASSO at λ_1_SE in at least four imputations: female BMI, male BMI, basal FSH, AMH, and female age. Variables such as menstrual regularity, infertility type, and education level were not stably selected and were therefore excluded. This stability-based selection suggests that spousal BMI and ovarian reserve markers constituted the most robust predictors within the available feature set.

### 3.3 Machine learning model performance evaluation

**Figure 2:**
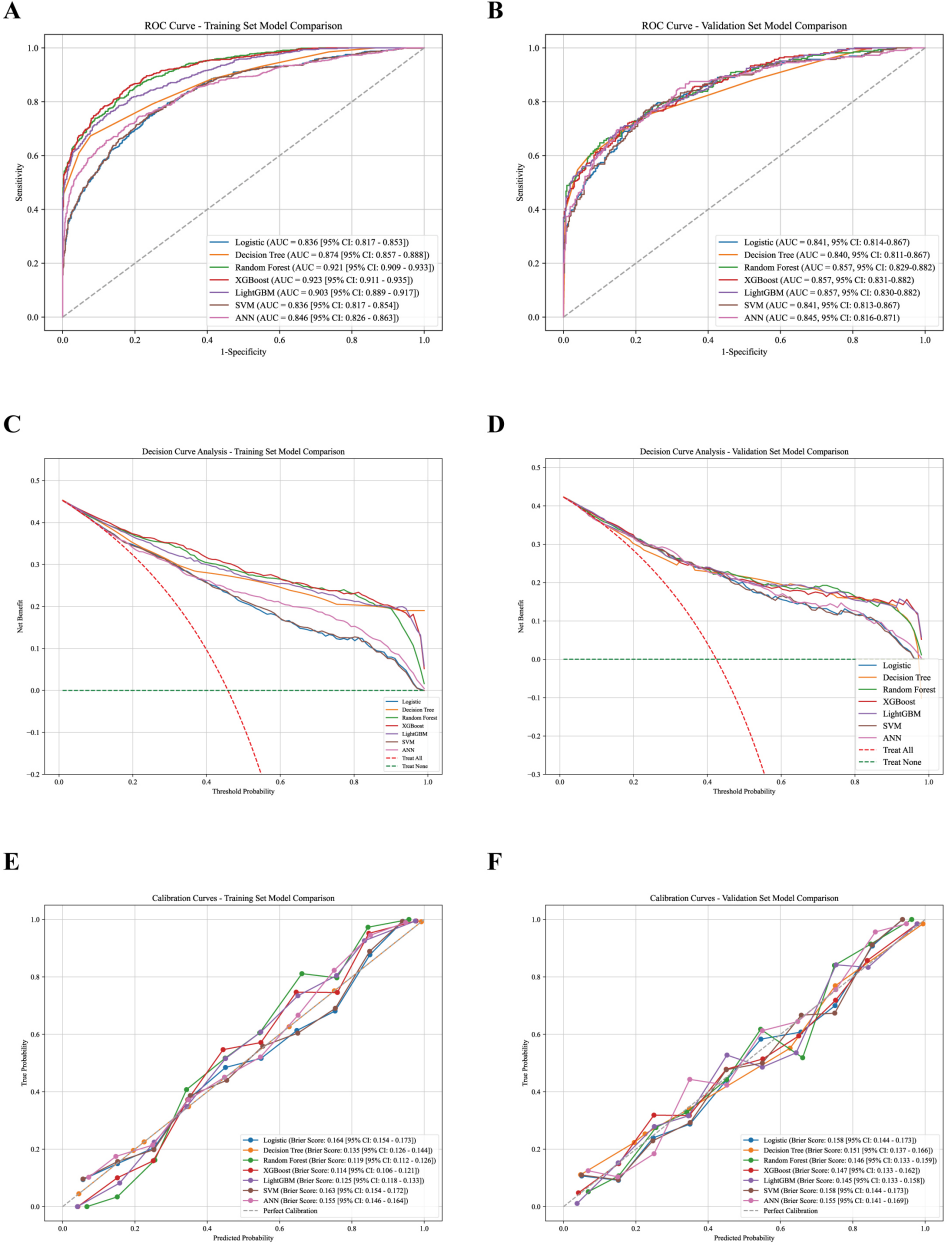
Performance evaluation of seven machine learning models in the training and validation sets. Receiver operating characteristic (ROC) curves for the training set **(A)** and validation set **(B)**. Decision curve analysis (DCA) for the training set **(C)** and validation set **(D)**. Calibration curves for the training set **(E)** and validation set **(F)**.

**Figure 3:**
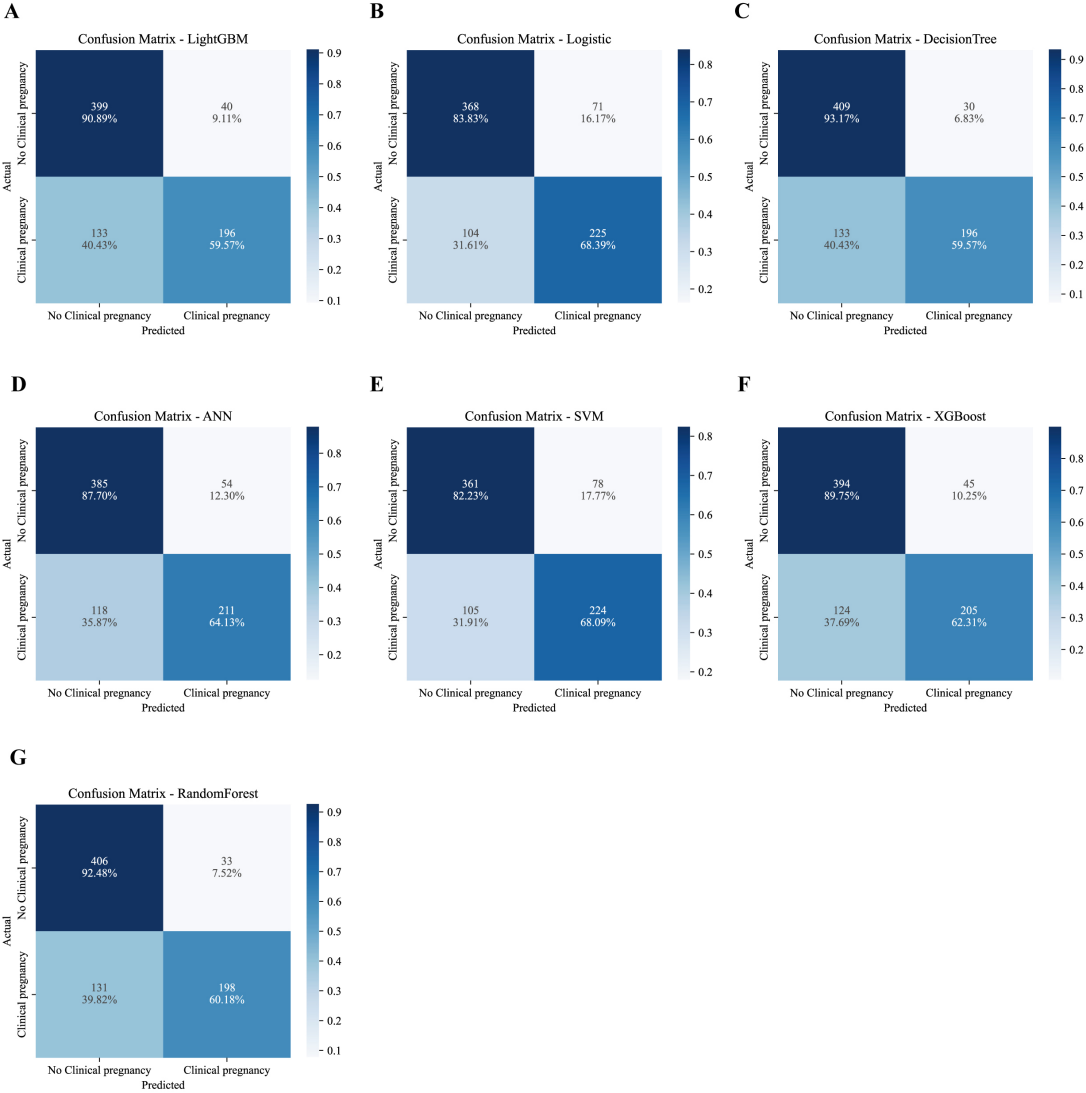
Confusion matrix heatmaps of machine learning models in the validation set. **(A)** LightGBM; **(B)** Logistic Regression; **(C)** XGBoost; **(D)** Random Forest; **(E)** Decision Tree; **(F)** Support Vector Machine (SVM); **(G)** Artificial Neural Network (ANN).

The predictive performance of the seven models in the training and validation sets is summarized in **Tables 2** and **Supplementary Table S3**, with ROC curves, calibration plots, and decision curve analyses shown in **Figure 2**, and confusion matrices presented in **Figure 3**. In the training set, ensemble models (Random Forest, XGBoost, and LightGBM) achieved higher AUCs than other algorithms (AUCs 0.903–0.923), whereas model performance became more comparable in the temporal validation set, with AUCs ranging narrowly from 0.840 to 0.857 across all models (**Table 2**, **Figure 2B**). LightGBM, XGBoost, and Random Forest demonstrated almost identical discriminative ability (all AUC = 0.857). DeLong tests confirmed that there were no statistically significant differences in AUC between LightGBM and Random Forest (P = 0.918), XGBoost (P = 0.985), or Logistic Regression (P = 0.067).

**Table 2:** Predictive performance of seven models in the validation set.

Model	AUC (95% CI)	Delong test P (vs.LightGBM)	Delong test P (vs.Logistic)	Accuracy	Precision	Sensitivity	Specificity	F1 Score	Kappa	PPV	NPV
Logistic	0.842 (0.814–0.867)	0.067	–	0.772	0.760	0.684	0.838	0.720	0.529	0.760	0.780
Decision Tree	0.840 (0.811–0.867)	0.027	0.877	0.788	0.867	0.596	0.932	0.706	0.549	0.867	0.755
Random Forest	0.857 (0.829–0.882)	0.918	0.090	0.787	0.857	0.602	0.925	0.707	0.547	0.857	0.756
XGBoost	0.857 (0.831–0.882)	0.985	0.075	0.780	0.820	0.623	0.898	0.708	0.537	0.820	0.761
LightGBM	0.857 (0.830–0.882)	–	0.067	0.775	0.831	0.596	0.909	0.694	0.523	0.831	0.750
SVM	0.841 (0.813–0.867)	0.051	0.430	0.762	0.742	0.681	0.822	0.710	0.508	0.742	0.775
ANN	0.845 (0.816–0.871)	0.163	0.469	0.776	0.796	0.641	0.877	0.710	0.531	0.796	0.765

AUC, Area Under the ROC Curve; PPV, Positive Predictive Value; NPV, Negative Predictive Value.

Beyond discrimination, LightGBM showed a balanced performance profile in the validation set, with an accuracy of 0.775, high specificity of 0.909 and moderate sensitivity (0.596). Calibration analysis suggested reasonable agreement between predicted and observed risks (Brier score = 0.145; **Figure 2F**), and decision curve analysis indicated net clinical benefit across a range of clinically plausible threshold probabilities (**Figure 2D**). Taken together, LightGBM was selected as the representative model for subsequent interpretability analyses due to its overall stability across discrimination, calibration, and clinical utility, rather than on statistically superior AUC alone.

Analysis of confusion matrices (**Figure 3**) further illustrated model behavior. LightGBM achieved a high true-negative rate (92.20%), reflecting strong specificity, while maintaining a sensitivity comparable to other models. From a clinical perspective, this tendency to limit false-positive predictions may be advantageous for avoiding overly optimistic prognostic assessments in couples with low likelihood of pregnancy.

### 3.4 Model interpretability analysis

**Figure 4:**
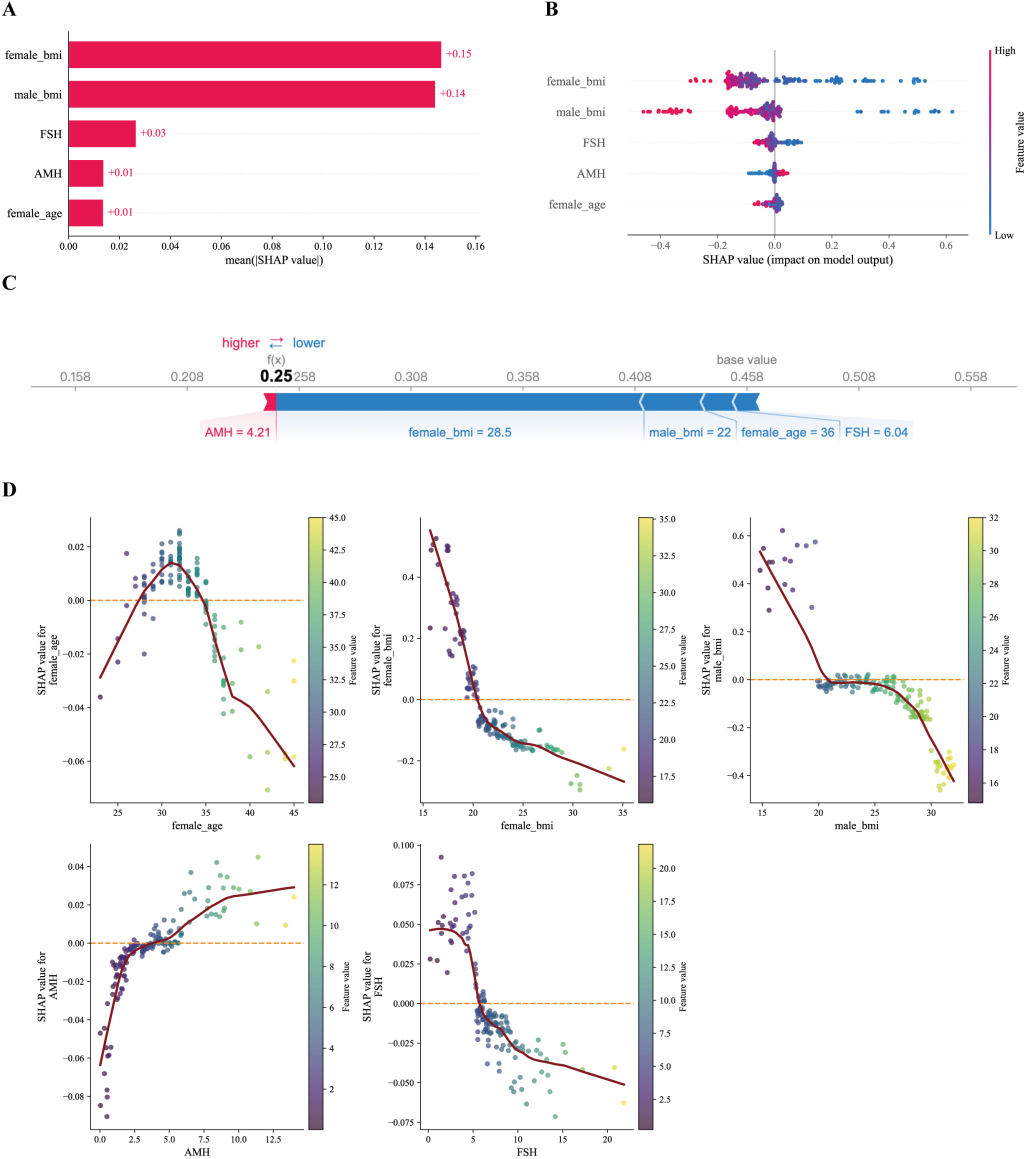
LightGBM model explanation by the SHAP method. **(A)** Bar chart of the all features. **(B)** Beeswarm plot. **(C)** Force plot for one non-pregnant patient. **(D)** SHAP dependency plot of features in the LightGBM model.

To enhance interpretability of the selected model, SHAP was applied to visualize the contribution of individual predictors to model output (**Figure 4**). The SHAP bar plot (**Figure 4A**), based on mean absolute SHAP values, indicated that male BMI and female BMI showed the highest average contributions within the fitted model and the available feature set, followed by basal FSH, AMH, and female age. The SHAP beeswarm plot (**Figure 4B**) further illustrated the direction of these associations: higher BMI values in either partner (red points) were predominantly located on the negative side of the x-axis, suggesting that higher BMI was associated with lower predicted probability of clinical pregnancy in the model output.

The SHAP force plot (**Figure 4C**) presents an illustrative individual case, showing how each feature contributed to shifting the prediction from the baseline toward a lower probability of pregnancy. In this example, elevated male BMI and older female age exerted negative contributions that outweighed the positive contribution of AMH. This visualization demonstrates how the model integrates multiple features to generate a personalized prediction, while reflecting model behavior rather than biological causation.

Finally, the SHAP dependence plots (**Figure 4D**) suggested nonlinear relationships between predictors and model output. Both female BMI and male BMI showed a threshold-like pattern: SHAP values remained relatively neutral within the lower range but declined sharply once BMI exceeded approximately the upper-normal range. AMH demonstrated a modest positive association at low-to-moderate levels, while higher FSH and increasing female age were associated with progressively negative SHAP values. These patterns reflect how the fitted model utilizes these predictors and should be interpreted as model-based associations rather than evidence of specific biological thresholds.

## 4 Discussion

This study developed and validated a prediction model for IVF/ICSI pregnancy outcomes in couples with male factor infertility using a single-center, large-sample retrospective cohort and the LightGBM algorithm. Compared with conventional logistic regression (**Supplementary Table S4**), ensemble tree–based methods may offer theoretical flexibility, although discrimination was comparable across models in our study. In the validation set, LightGBM showed an AUC of 0.857 and a specificity of 90.9%. SHAP-based interpretation suggested that, within the fitted model and conditional on the available predictors, spousal body mass index (BMI) exhibited relatively larger contributions to model predictions than traditional ovarian reserve indicators such as basal FSH, AMH, and female age. Importantly, these findings reflect model-based associations rather than causal effects. Nevertheless, the results suggest that, in the clinical context of impaired sperm quality, couple-level metabolic characteristics may represent an underappreciated dimension in prognostic assessment. These findings complement existing frameworks that traditionally emphasize ovarian reserve (32, 33).

This observation is biologically plausible within the pathophysiological context of male factor infertility and may generate hypotheses for future research. Based on existing literature, we propose a conceptual “two-hit” hypothesis as a possible interpretive framework rather than a conclusion supported directly by our data. First, patients with oligozoospermia, asthenozoospermia, or teratozoospermia frequently show increased sperm DNA fragmentation and aberrant epigenetic alterations, and obesity-associated systemic oxidative stress in males may further exacerbate these abnormalities (34, 35). Although ICSI can bypass physical barriers to fertilization, it does not rectify molecular defects carried by sperm, which may lead to embryos with reduced developmental competence, constituting the first hit (36). Second, when female BMI exceeds a threshold, obesity-related chronic low-grade inflammation may alter endometrial gene expression and compromise receptivity and decidualization, representing the second hit (37–39). Importantly, the present study did not directly measure sperm DNA fragmentation, epigenetic alterations, or endometrial receptivity. Therefore, this conceptual framework should be regarded as hypothesis-generating and requires validation in future mechanistic and experimental studies.

Although multiple imputation was used to address missing data, performance metrics and statistical tests were primarily derived from pooled predicted probabilities rather than from fully Rubin-combined estimates across imputations. This approach may underestimate uncertainty because between-imputation variability is not fully propagated. However, given the very low proportion of missingness (<5% for all predictors), the impact of this limitation is likely modest. Future studies with higher levels of missingness should consider fully nested bootstrap–imputation procedures to provide more rigorous uncertainty quantification.

In the SHAP dependence plots, a gradual decline in SHAP values was observed as BMI increased, with a more apparent decrease beyond approximately 24–25 kg/m². This apparent threshold should be interpreted with caution for several reasons and does not imply a clinically actionable cutoff or an intervention threshold. First, the value was derived from visual inspection of SHAP-based plots and reflects model behavior under correlated predictors rather than a clinically or statistically validated boundary. We did not apply formal methods for threshold identification, such as spline-based regression, uncertainty-aware partial dependence analysis, or analyses based on prespecified BMI categories. Second, the observed value closely corresponds to the Chinese definition of overweight (BMI ≥24 kg/m²), indicating that this pattern may partly reflect population-specific characteristics. Therefore, the generalizability of this threshold beyond the present cohort remains uncertain and warrants validation in external populations using alternative BMI classification standards.

These findings may have potential clinical implications, but they should be interpreted with appropriate caution. Rather than advocating a change in clinical practice, our results highlight the possible value of considering couple-level metabolic health alongside traditional ovarian-centered assessments (40). In current practice, clinical efforts often focus on optimizing ovarian stimulation to increase oocyte yield. Our model suggests that metabolic factors may contribute to prognostic stratification and may be useful during patient counseling. However, whether targeted preconception interventions, such as weight reduction—particularly in the male partner—lead to improved ART outcomes remains uncertain and requires confirmation in prospective interventional studies. Therefore, BMI should be regarded as a potentially informative predictive marker in this dataset rather than a basis for mandatory treatment delay or universal prioritization of weight intervention (41).

## 5 Conclusion

A LightGBM-based model demonstrated reasonable predictive performance for IVF/ICSI pregnancy outcomes in couples with male factor infertility, with relatively high specificity in the validation set. Model interpretation suggested that, within the fitted model and available feature set, couple-level metabolic characteristics were associated with predicted outcomes alongside traditional ovarian reserve markers. These findings represent predictive associations rather than causal effects. BMI may serve as a potentially informative prognostic feature for counseling and risk stratification in this population, while the clinical benefit of targeted metabolic interventions requires confirmation in prospective and interventional studies.

## Data Availability

The raw data supporting the conclusions of this article will be made available by the authors, without undue reservation.
