# Supplementary material for: Machine learning–based prediction of IVF/ICSI outcomes in male factor infertility highlighting couple-level BMI
Source: Front Endocrinol (Lausanne). 2026 Feb 10;17:1772106. doi: 10.3389/fendo.2026.1772106 (PMC12929144; doi:10.3389/fendo.2026.1772106)
Supplement: Supplementary file 1 [file DataSheet1.docx]

**Table S1. Missingness Proportion of Study Variables in the Training and Validation Cohorts**

| Variable | training set | validation set |
| --- | --- | --- |
| Outcome | 0 | 0 |
| female_education | 0 | 0 |
| male_education | 0 | 0 |
| infertility_type | 0 | 0 |
| menstrual_pattern | 0 | 0 |
| female_age | 0 | 0 |
| male_age | 0 | 0 |
| female_bmi | 0 | 0 |
| male_bmi | 0 | 0 |
| infertility_duration | 0.0039 | 0.0039 |
| AMH | 0.0011 | 0.0013 |
| FSH | 0.0045 | 0.0052 |
| LH | 0.0061 | 0.0078 |
| PRL | 0.0173 | 0.0195 |
| E2 | 0.0056 | 0.0013 |
| T | 0.0045 | 0.0026 |
| P | 0.0067 | 0.0065 |
| afc_total | 0.0006 | 0.0013 |

**Table S2. Imputation methods by variable in the training set**

| Variable | Method |
| --- | --- |
| Outcome | - |
| female_education | - |
| male_education | - |
| infertility_type | - |
| menstrual_pattern | - |
| female_age | - |
| male_age | - |
| female_bmi | - |
| male_bmi | - |
| infertility_duration | pmm |
| AMH | pmm |
| FSH | pmm |
| LH | pmm |
| PRL | pmm |
| E2 | pmm |
| T | pmm |
| P | pmm |
| afc_total | pmm |

Pmm: predictive mean matching

**Table S3. Predictive performance of seven models in the training set**

| Model | AUC (95% CI ) | Delong test P (vs.LightGBM) | Delong test P (vs.Logistic) | Accuracy | Precision | Sensitivity | Specificity | F1 Score | Kappa | PPV | NPV |
| --- | --- | --- | --- | --- | --- | --- | --- | --- | --- | --- | --- |
| Logistic | 0.836 (0.817–0.853) | < 0.001 | - | 0.751 | 0.743 | 0.699 | 0.796 | 0.720 | 0.497 | 0.743 | 0.758 |
| Decision Tree | 0.874 (0.857–0.888) | < 0.001 | < 0.001 | 0.809 | 0.881 | 0.673 | 0.923 | 0.763 | 0.607 | 0.881 | 0.770 |
| Random Forest | 0.921 (0.909–0.933) | < 0.001 | < 0.001 | 0.824 | 0.911 | 0.682 | 0.944 | 0.780 | 0.638 | 0.911 | 0.778 |
| XGBoost | 0.923 (0.911–0.935) | < 0.001 | < 0.001 | 0.826 | 0.889 | 0.710 | 0.925 | 0.789 | 0.645 | 0.889 | 0.790 |
| LightGBM | 0.903 (0.889–0.917) | - | < 0.001 | 0.815 | 0.894 | 0.676 | 0.932 | 0.770 | 0.620 | 0.894 | 0.773 |
| SVM | 0.836 (0.817–0.854) | < 0.001 | 0.5334 | 0.756 | 0.747 | 0.705 | 0.799 | 0.725 | 0.506 | 0.747 | 0.762 |
| ANN | 0.846 (0.826–0.863) | < 0.001 | 0.0083 | 0.774 | 0.812 | 0.657 | 0.872 | 0.727 | 0.537 | 0.812 | 0.751 |

AUC: Area Under the ROC Curve; PPV: Positive Predictive Value; NPV: Negative Predictive Value;

Training-set performance is reported only for completeness and was not used for model comparison or inference.

**Table S4. Logistic regression results for predictors of clinical pregnancy in the training and validation sets**

| Variables | Training set  OR (95% CI) | P-value | Validation set  OR (95% CI) | P-value |
| --- | --- | --- | --- | --- |
| female_bmi | 0.732(0.703-0.762) | <0.001 | 0.726(0.679-0.775) | <0.001 |
| male_bmi | 0.787(0.763-0.811) | <0.001 | 0.755(0.719-0.792) | <0.001 |
| female_age | 0.965(0.936-0.994) | 0.019 | 0.984(0.936-1.034) | 0.516 |
| AMH | 1.024(0.990-1.060) | 0.173 | 1.000(0.948-1.055) | 0.992 |
| FSH | 0.892(0.853-0.933) | <0.001 | 0.910(0.843-0.982) | 0.015 |

**Figure S1.**


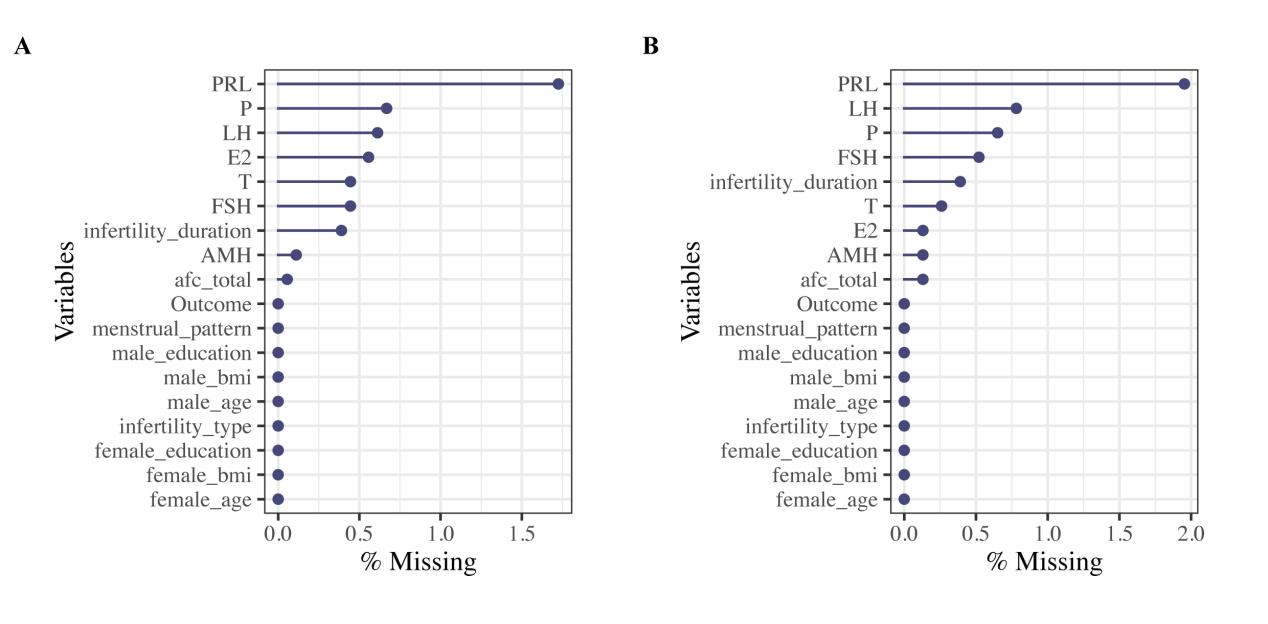


**Figure S1.** Distribution of missing data across variables in the training and validation sets.

(A) Percentage of missing values for each variable in the training set.

(B) Percentage of missing values for each variable in the validation set.

**Figure S2.**

**
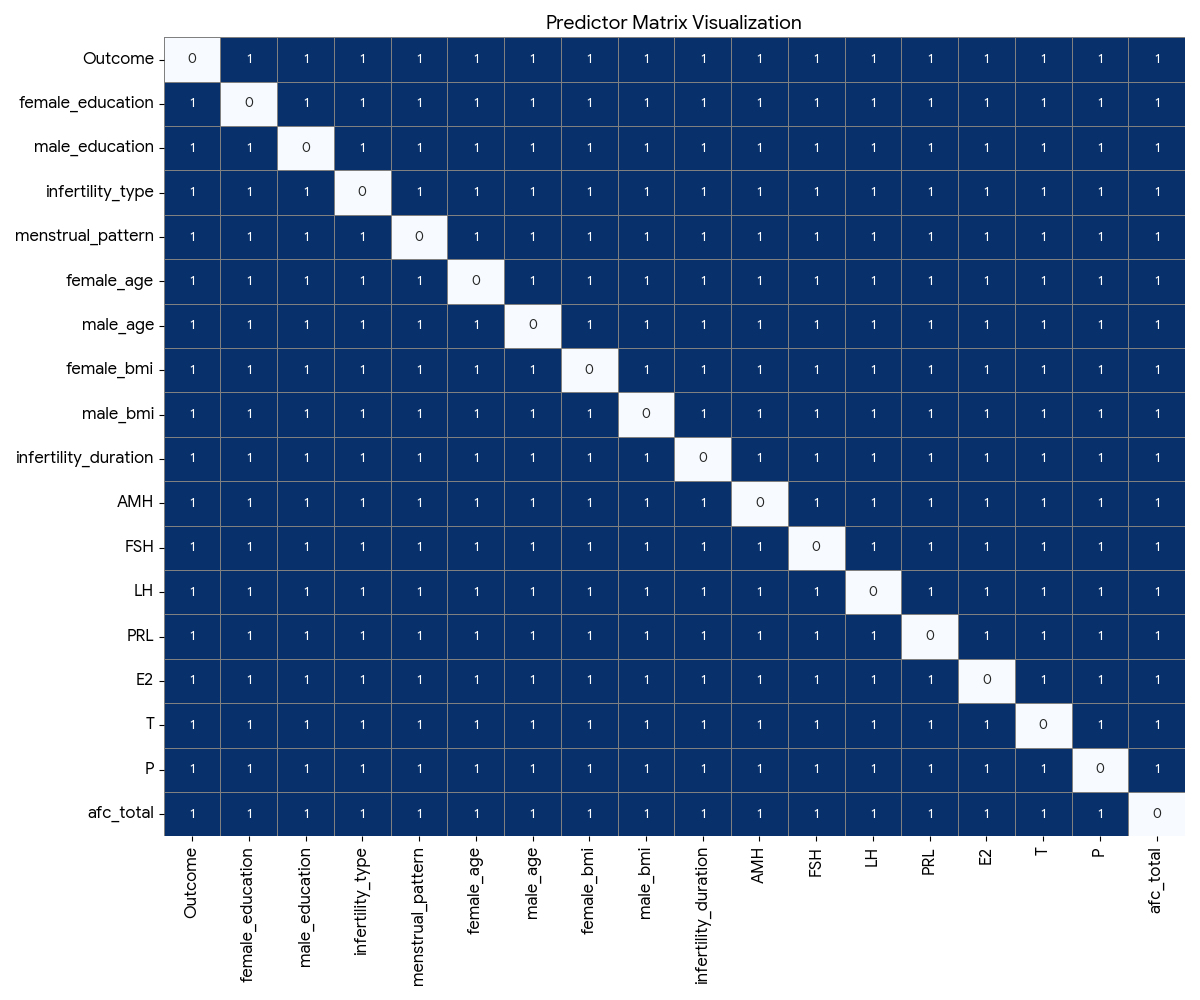
**

**Figure S2. PredictorMatrix used for MICE in the training set**
